# Dermoscopic Predictors of Tumor Thickness in Cutaneous Melanoma: A Retrospective Analysis of 245 Melanomas

**DOI:** 10.5826/dpc.1103a59

**Published:** 2021-05-20

**Authors:** Enrique Rodríguez-Lomba, Belén Lozano-Masdemont, Lula María Nieto-Benito, Elisa Hernández de la Torre, Ricardo Suárez-Fernández, José Antonio Avilés-Izquierdo

**Affiliations:** 1 Department of Dermatology, Hospital General Universitario Gregorio Marañón, Madrid, Spain; 2 Department of Dermatology, Hospital Universitario de Móstoles, Madrid, Spain

**Keywords:** melanoma, Breslow index, tumor thickness, dermoscopy, dermatoscopy, epiluminescence microscopy

## Abstract

**Introduction:**

The literature regarding the association of dermoscopic structures with Breslow thickness in melanoma is scarce, limited to small case series, and mostly outdated.

**Objective:**

This study determined the dermoscopic patterns, colors and structures that are associated with melanoma in situ, thin melanomas (<0.8 mm) and thick melanomas potentially requiring sentinel lymph node biopsy according to current guidelines (≥0.8 mm).

**Methods:**

A retrospective evaluation of 245 dermoscopic images of primary cutaneous melanoma located on the trunk or limbs was performed by consensus of 2 dermoscopists.

**Results:**

Red-pink, blue-gray and white color, blue-white veil, shiny white streaks, irregular vessels, blue-black pigmentation, milky red areas, pseudolacunae, ulceration and rainbow pattern were associated with thickness ≥0.8 mm, whereas atypical pigmented network, regression and hypopigmented areas were significantly associated with early melanomas.

**Limitations:**

This is a retrospective study performed in a single institution. Melanomas of special sites were excluded from our evaluation. Dermoscopy is based on subjective evaluations that depend largely on the observers’ experience.

**Conclusions:**

The identification of certain dermoscopic structures and colors might help in the discrimination between thin and thick melanomas.

## Introduction

The literature regarding the association of patterns, colors and dermoscopic structures with Breslow thickness in melanoma is scarce, limited to small case series, and mostly based on outdated nomenclature [1–5]. Certain specific dermoscopic structures could help in the early identification of melanomas with tumor thickness ≥0.8 mm, potentially eligible for sentinel lymph node biopsy according to current guidelines. On the other hand, one author [6] recently criticized the established recommendation of re-excision of clinical safety margins in thin melanomas that have already been completely excised. According to them, a “personalized excision” approach would be preferable and should be included in future guidelines [6]. Dermoscopy could be useful in the identification of sharply confined melanomas with a high probability of tumor thickness <0.8 mm, allowing us to excise them in a single surgical procedure with adjusted margins. The aim of this study was to determine the dermoscopic colors and structures that are associated with both early melanomas (<0.8 mm) and thick melanomas (≥0.8 mm).

## Materials and Methods

A retrospective evaluation of 245 dermoscopic images of primary cutaneous melanoma was performed. All the dermoscopic images were collected from the database of the Melanoma Unit in our department. These were obtained using a digital microscopy system comprising a DermLite Photo II Pro HR dermoscopy lens [3Gen] on an E-420 camera (Olympus). The lesion diameter had to be small enough to fit in the whole picture in order to qualify for inclusion. We excluded cases without histopathological confirmation, cases with melanoma metastases, as well as primary melanomas of special sites (facial, acral, nail, genital or mucosal melanoma). Images with thick hair density, blood or scales that impeded an adequate dermoscopic evaluation were also excluded. Clinical and histopathological data was obtained from patients’ records and included age at diagnosis, sex, anatomical location of the tumor, and tumor diameter, palpability, and Breslow thickness.

A list of the dermoscopic criteria established by previous publications was evaluated by consensus of 2 blinded expert dermoscopists (J.A.A.I., E.R.L). The following dermoscopic features were analyzed: colors (light brown, dark brown, black, blue-gray, red-pink, white), asymmetry of color and structures, atypical pigmented network, irregular globules, streaks, irregular blotches, shiny white streaks, negative pigment network, blue-white veil, hypopigmented areas, prominent skin markings, structureless brown areas, blue-black pigmentation, milky red areas, rainbow pattern, pseudolacunae, ulceration, and irregular vessels.

Data were analyzed using SPSS version 22.0 (32-bits edition). Univariate analysis for qualitative variables was performed using Pearson’s chi-square test and Fisher’s exact test. A P value less than .05 was considered statistically significant. Sensitivity, specificity, positive predictive value (PPV) and negative predictive value (NPV) were calculated using 2×2 contingency tables. The odds ratio (OR) was calculated for all variables with a confidence interval of 95% (95% CI).

## Results

A total of 245 melanomas were analyzed. Three subtypes were initially defined: melanoma *in situ* (intraepidermal), thin melanomas (Breslow thickness <0.8 mm), and thick melanomas (Breslow thickness ≥0.8 mm). The number of cases in each of these groups was 52 (21.2%), 98 (40.0%), and 95 (38.7%), respectively. The mean Breslow thickness was 0.98 mm (range, 0–9.00 mm). The median Breslow thickness was 0.60 mm, and only 23 melanomas (9.3%) had a Breslow thickness larger than 1.5 mm.

Clinical and epidemiological features are presented in Table 1. Tumor diameter and palpability were the only clinical variables that showed statistically significant differences (P = .001 for both). All *in situ* melanomas were non-palpable. Less than half of the <0.8 mm melanomas were clinically raised (n = 46; 46.9%), while most of the ≥0.8 mm melanomas were palpable (n = 86; 90.5%). Thick melanomas were more frequently larger than 10 mm (73.6%) than were thin melanomas (50.0%) and melanoma *in situ* (48.1%). There were no significant differences in age at diagnosis (mean ages, 59.13, 57.55 and 59.19 years, respectively) or sex distribution among the groups. Melanoma *in situ* and thin melanomas were slightly more frequent in women (53.8% and 53.1%, respectively), while thick melanomas were more prevalent in men (53.7%). Most of the melanomas were located in areas of intermittent sun exposure (n = 225; 91.8%) due to the exclusion criteria in our study design.

Table 2 summarizes the frequencies of colors and dermoscopic structures within each group. Light brown color was more frequent in early melanomas (melanoma *in situ* and thin melanoma), while blue-gray, red-pink or white color was more commonly observed in thick melanomas (P <.05). The presence of three or more colors showed no significant difference between groups. Early melanomas presented a higher frequency of atypical pigmented network, regression and hypopigmented areas. Shiny white streaks, blue-white veil, blue-black pigmentation, milky red areas, rainbow pattern, pseudolacunae, ulceration, irregular vessels and polymorphous vascular pattern were more frequent in thick melanomas (P <.05). No significant differences were observed in color or structure asymmetry between the three groups.

Two cohorts were grouped for the calculation of sensitivity, specificity, PPV, NPV and OR: early melanomas (n = 150) and thick melanomas (n = 95). Results are presented in Table 3. The presence of three or more colors presented the highest sensitivity (88.4%) for a thickness ≥0.8 mm, although most of the criteria showed sensitivity values lower than 50%. Most of the criteria showed higher than 80% specificity for ≥0.8 mm tumor thickness. The highest specificity was obtained for pseudolacunae (98.0%), ulceration (96.7%) and rainbow pattern (95.3%). PPV was highest for ulceration (87.5%) and pseudolacunae (88.5%), whereas the other features showed low values. NPV was lower than 80% for all dermoscopic features. The following colors and structures were associated with melanoma thickness ≥0.8 mm: red-pink (OR = 4.641), blue-gray (OR = 3.743), white (OR = 3.168), blue-white veil (OR = 8.446), shiny white streaks (OR = 2.913), irregular vessels (OR = 3.796), blue-black pigmentation (OR = 4.149), milky red areas (OR = 4.668), pseudolacunae (OR = 15.653), ulceration (OR = 16.917) and rainbow pattern (OR = 7.296). On the other hand, atypical pigmented network (OR = 5.505), regression (OR = 0.408) and hypopigmented areas (OR = 0.372) were significantly associated with early melanomas.

## Discussion

Argenziano et al [1] reported for the first time in 1997 the differences of colors and dermoscopic structures in a case series of 72 melanomas (41 thin melanomas <0.75 mm, 31 thick melanomas ≥0.75 mm). The authors reported a higher frequency of pigment network, radial streaming and white scar-like areas in thin melanomas, whereas gray-blue areas, structural asymmetry and a vascular pattern were more frequent in thick melanomas. A significant association between the presence of a pigment network and thin melanomas was noted, as well as gray-blue areas and a vascular pattern in thick melanomas. These findings were confirmed in a study of 84 melanomas by Stante et al [2].

A few attempts have been made to reliably predict tumor thickness by dermoscopy, such as the clinical-dermoscopic algorithm published in 1999 by Argenziano et al [3]. The authors reported that the combination of palpability, tumor diameter ≥15 mm, pigment network, gray-blue areas, and atypical vascular pattern increased the prediction accuracy by 14% compared to palpability alone and 9% compared to dermoscopy alone. Others have tried to apply well-known dermoscopic algorithms to predict tumor thickness. The ABCD rule had an adequate performance in predicting tumor thickness in a series of 84 cutaneous melanomas when a total dermatoscopy score (TDS) cut-off of 6.80 was applied [4]. Despite the initially promising results of the preoperative assessment of melanoma thickness by dermoscopy, few studies of this approach have been published since then.

Nomenclature in dermoscopy has expanded over the past decade, and the impact of some dermoscopic structures has not yet been studied. Melanoma guidelines have been updated, and 0.8 mm is now considered the cut-off between T1a and T1b melanomas [7]. The present study expands on this literature and updates it with updated nomenclature. In addition, our study had a bigger sample than previous studies, and we investigated a larger variety of dermoscopic structures.

The only clinical features that reached statistical significance were tumor size ≥10 mm, a slightly lower cut-off than 15 mm as previously reported [3], and palpability. Although *in situ* melanomas were all non-palpable and more than 90% of the >0.8 mm thick melanomas were clearly palpable, 46.9% of the early invasive melanomas (<0.8 mm) were also palpable. The analysis of this single parameter would have classified these thin melanomas as thick when they were not, and on the contrary, 9.5% of thick melanomas would have been wrongly classified as thin.

Table 4 presents the colors and structures associated with melanomas <0.8 mm and ≥0.8 mm. Thin melanomas were associated with light brown color whereas thick melanomas were associated not only with blue-gray color, but also red-pink and white. However, the clinical and dermoscopic evaluation of colors must take into account the area of examination, both histologically and dermoscopically. It is not uncommon for melanomas, especially superficial-spreading subtypes, to present areas of different thickness throughout their width. Thick melanomas may show light brown areas in their periphery, and blue areas in the thicker center that must always be considered.

**Figure 1:**
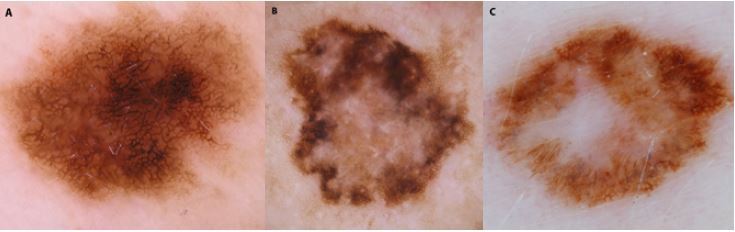
Dermoscopic predictors of thin melanoma (<0.8 mm tumor thickness). (A) Atypical pigmented network. (B) Regression. (C) Hypopigmented areas.

**Figure 2:**
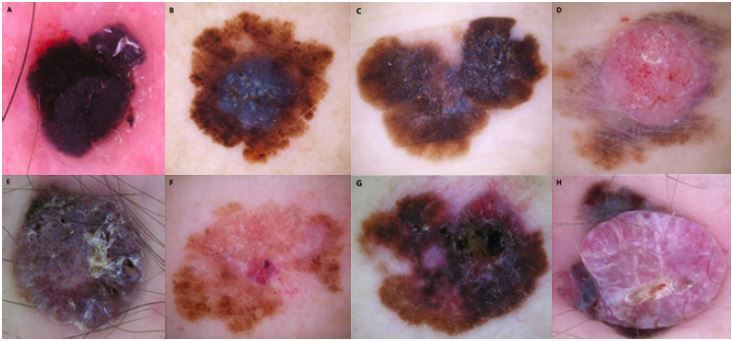
Dermoscopic predictors of thick melanoma (≥0.8 mm tumor thickness). (A) Blue-black pigmentation. (B) Blue-white veil. (C) Shiny white streaks. (D) Irregular vessels. (E) Rainbow pattern. (F) Milky red areas. (G) Ulceration. (F) Pseudolacunae.

No statistically significant differences were observed regarding asymmetry of color or structures, unlike a previous study based on digital analysis [8]. This discrepancy might be related to a more rigid interpretation of asymmetry by computerized algorithms than the human eye. Atypical pigmented network, regression and hypopigmented areas were associated with early melanomas here (Figure 1) as in previous reports [1, 2]. Ulceration, pseudolacunae, blue-white veil, milky red areas, irregular vessels, blue-black pigmentation and shiny white streaks were associated with a melanoma tumor thickness ≥0.8 mm (Figure 2). The latter has already been reported to be associated with thicker melanomas in a case series of 144 melanomas [9]. The evaluation of dermoscopic structures should consider the same principles mentioned previously in color evaluation.

Mun et al [10] recently reported the dermoscopic differences between thin and thick acral melanomas (<2 vs. >2 mm) in a cohort of 75 cases. The authors concluded that blue (OR = 7.09), white (OR = 5.04), atypical vessels (OR = 34.58), blue-white veil (OR = 9.60) and ulceration (OR = 5.08) were associated with thick acral melanomas. While our study specifically excluded acral melanomas, most of our results are consistent with theirs.

The combination of dermoscopy and other imaging techniques such as optical coherence tomography, multispectral imaging and high-frequency ultrasonography could further enhance the preoperative assessment of melanoma patients towards “personalized medicine”. Together with palpability and these specific dermoscopic findings, they could allow a fairly precise tumor thickness prediction. However, caution is recommended before the excision of early melanomas in a single surgical procedure. To date, clinical guidelines do not have a recommendation for this approach, and further evidence is required before standardization. It should be limited to cases with a high malignancy suspicion, and not done in doubtful cases, in order to minimize the risk of causing unnecessary, large scars in benign lesions. The benefits and risks of performing a single-step surgery on a patient need to be adequately addressed before proceeding.

Limitations of our study are its retrospective nature and single-institution design. Melanomas of special sites were excluded from our evaluation. Dermoscopy is based on subjective evaluations that depend largely on the observers’ experience. Larger prospective studies focusing on the reliability of the combination of certain colors and structures are required to confirm and validate our findings.

## Conclusions

Certain dermoscopic structures and colors might help in the discrimination between thin and thick melanomas. Although none of them are entirely specific to either group, the combination of more than one of them in a single lesion increases the probability of an adequate tumor thickness prediction. For example, pigmented lesions presenting palpable blue-white veil, milky red areas and ulceration are unlikely to be early melanomas. On the other hand, lesions presenting an atypical pigmented network and small foci of regression without any other dermoscopic features are very unlikely to be >0.8 mm thickness. The reliability of the combination of certain dermoscopic colors and structures is to be determined, and should be the subject of future studies.

## Figures and Tables

**Table 1:** Clinical and Epidemiological Features, by Tumor Thickness

Feature	Melanoma in situ (n = 52)	Melanoma <0.8 mm (n = 98)	Melanoma ≥0.8 mm (n = 95)	P
Mean age, y	59.13	57.55	59.19	NS
≥65 y, n (%)	23 (44.2)	43 (43.9)	43 (45.3)	NS
Male:Female, n (%)	24:28 (46.2:53.8)	46:52 (46.9:53.1)	51:44 (53.7:46.3)	NS
Palpability, n (%)	0 (0)	46 (46.9)	86 (90.5)	.001
Tumor diameter ≥10 mm, n (%)	25 (48.1)	49 (50.0)	70 (73.6)	.001
Tumor location, n (%)				NS
Chronic exposure	5 (9.6)	7 (7.1)	2 (2.1)	
Intermittent exposure	45 (86.5)	89 (90.8)	91 (95.8)	
Non-exposed areas				
	2 (3.8)	2 (2.0)	2 (2.1)	

NS = Not significant.

**Table 2:** Frequencies of Colors and Dermoscopic Structures, by Tumor Thickness

Feature	Melanoma in situ (n = 52)	Melanoma <0.8 mm (n =98)	Melanoma ≥0.8 mm (n = 95)	P

Color				
Light brown	50 (96.2)	93 (94.9)	78 (82.1)	.003

Dark brown	49 (94.2)	92 (93.9)	79 (83.2)	.240

Black	34 (65.4)	47 (48.0)	55 (57.9)	.104

Blue-gray	22 (42.3)	58 (59.2)	77 (81.1)	.000

Red-pink	6 (11.5)	22 (22.4)	49 (51.6)	.000

White	6 (11.5)	23 (23.5)	41 (43.2)	.000
Three or more colors	42 (80.8)	81 (82.7)	84 (88.4)	.383

Atypical pigmented network	36 (69.2)	41 (41.8)	33 (34.7)	.000

Irregular globules	22 (42.3)	51 (52.0)	37 (38.9)	.172

Irregular blotches	22 (42.3)	54 (55.1)	40 (42.1)	.139

Regression	21 (40.4)	47 (48.0)	24 (25.3)	.004

Shiny white streaks	9 (17.3)	35 (35.7)	52 (54.7)	.000

Hypopigmented areas	8 (15.4)	15 (15.3)	6 (6.3)	.104

Streaks	7 (13.5)	23 (23.5)	18 (18.9)	.333

Prominent skin markings	6 (11.5)	7 (7.1)	5 (5.3)	.376

Structureless brown areas	5 (9.6)	13 (13.3)	15 (15.8)	.576

Blue-white veil	4 (7.7)	17 (17.3)	55 (57.9)	.000

Negative pigment network	4 (7.7)	7 (7.1)	7 (7.4)	.992

Irregular vessels	4 (7.7)	21 (21.4)	41 (43.2)	.000

Blue-black pigmentation	3 (5.8)	5 (5.1)	18 (18.9)	.003

Milky red areas	2 (3.8)	14 (14.3)	34 (35.8)	.000

Ulceration	0 (0)	4 (4.1)	35 (36.8)	.000

Rainbow pattern	0 (0)	6 (6.1)	25 (26.3)	.000

Pseudolacunae	0 (0)	3 (3.1)	23 (24.2)	.000

**Table 3:** Diagnostic Accuracy of Colors and Dermoscopic Structures for Tumor Thickness ≥0.8 mm

Feature	Sens.	Spec.	PPV	NPV	OR (95% CI)

Color					
Red-pink	51.6	81.3	63.6	72.6	4.641 (2.611–8.242)

Blue-gray	46.7	81.1	49.0	79.5	3.743 (2.044–6.853)

White	43.2	80.7	58.6	69.1	3.168 (1.785–5.626)

Black	57.9	46.0	40.4	63.3	1.123 (0.632–1.965)

Dark brown	83.2	6.0	35.9	36.0	0.315 (0.133–0.740)

Light brown	82.1	4.7	35.3	29.2	0.225 (0.081–0.562)
Three or more colors	88.4	18.0	40.6	71.1	1.676 (0.789–3.563)

Asymmetry of colors	78.9	22.7	39.3	63.0	1.099 (0.589–2.051)

Asymmetry of structures	78.9	16.0	37.3	54.5	0.714 (0.370–1.380)

Ulceration	36.8	96.7	87.5	70.7	16.917 (6.326–45.265)

Pseudolacunae	24.2	98.0	88.5	67.1	15.653 (4.549–53.854)

Blue-white veil	57.9	86.0	72.4	76.3	8.446 (4.568–15.67)

Rainbow pattern	26.3	95.3	78.1	67.1	7.296 (3.018–17.687)

Milky red areas	35.8	89.3	68.0	68.7	4.668 (2.395–9.096)

Blue-black pigmentation	18.9	94.7	69.2	64.8	4.149 (1.724–9.983)

Irregular vessels	43.2	83.3	62.1	69.8	3.796 (2.102–6.855)

Shiny white streaks	54.7	70.7	54.2	71.1	2.913 (1.702–4.973)

Structureless brown areas	15.8	88.0	45.5	62.3	1.375 (0.656–2.880)

Negative pigment network	7.4	92.7	38.9	61.2	1.005 (0.376–2.690)

Streaks	18.9	80.0	37.5	60.9	0.935 (0.488–1.792)

Irregular blotches	42.1	49.3	34.5	57.4	0.708 (0.422–1.189)

Irregular globules	38.9	51.3	33.6	57.0	0.673 (0.399–1.134)

Prominent skin markings	5.3	91.3	27.8	60.4	0.585 (0.202–1.699)

Atypical pigmented network	34.7	48.7	30.0	54.1	0.505 (0.295–0.856)

Regression	25.3	54.7	26.1	53.6	0.408 (0.238–0.717)

Hypopigmented areas	6.3	84.7	20.7	58.8	0.372 (0.149–0.950)

Sens. = sensitivity; Spec. = specificity; PPV = positive predictive value; NPV = negative predictive value; OR = odds ratio; CI = confidence interval.

**Table 4:** Significant Associations Between Specific Dermoscopic Features and Breslow Thickness

	Melanoma <0.8 mm	Melanoma ≥0.8 mm
Colors	Light brown	Red-pink Blue-gray White
Dermoscopic features	Atypical pigmented network Regression Hypopigmented areas	Blue-white veil Shiny white streaks Irregular vessels Blue-black pigmentation Milky red areas Rainbow pattern Pseudolacunae Ulceration
